# Supplementary figures and images for: Drosophila Topoisomerase 3β binds to mRNAs in vivo, contributes to their localization and stability, and counteracts premature aging
Source: PLoS One. 2025 Feb 11;20(2):e0318142. doi: 10.1371/journal.pone.0318142 (PMC12140118; doi:10.1371/journal.pone.0318142)

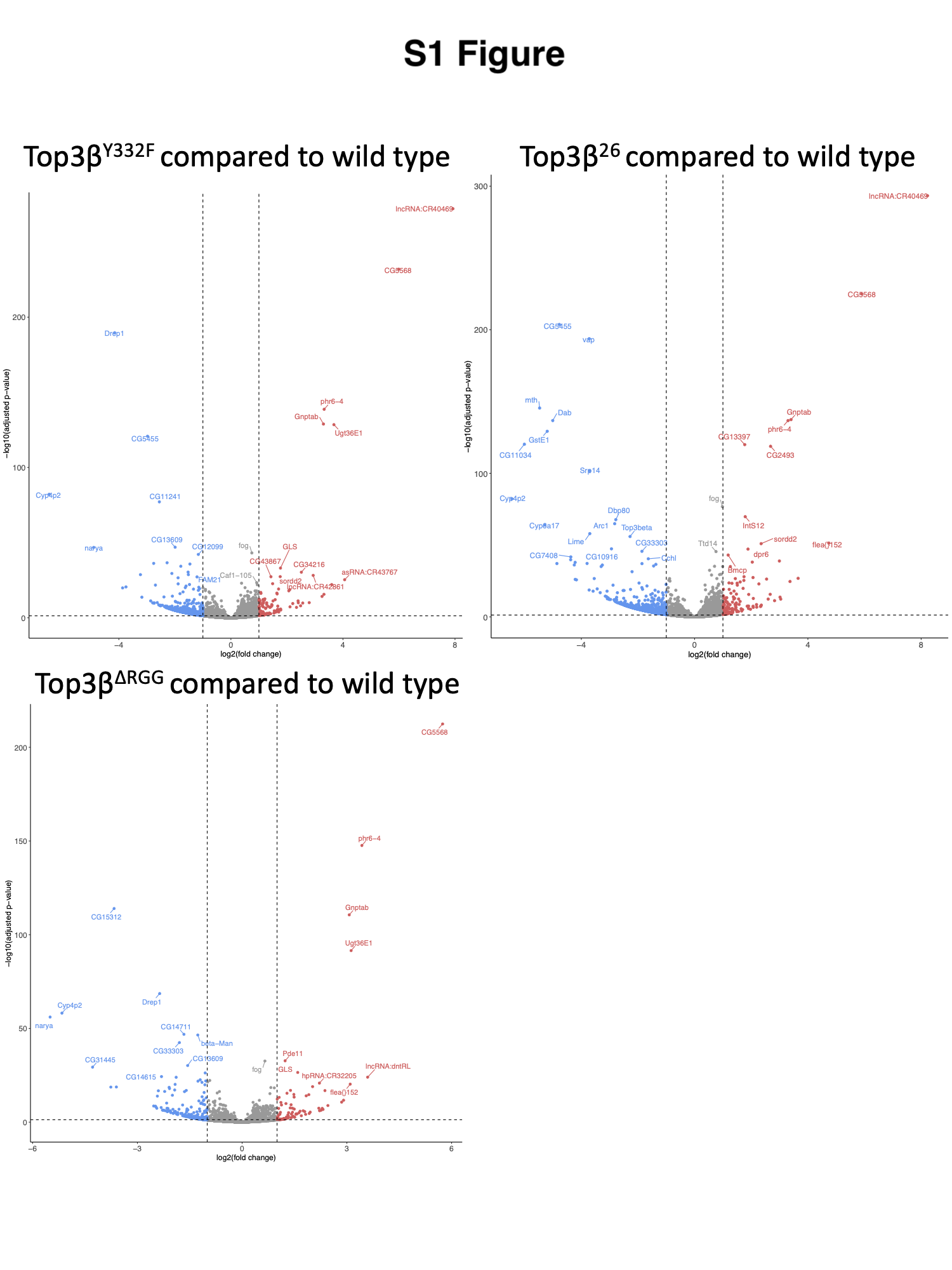

Supplement: S1 Fig — Volcano plots showing the effect of three Top3𝛽 mutations on the transcript levels in 0–2 hrs old Drosophila embryos. Adjusted p values are plotted against differences in transcript abundance. The logarithmic scales used for both axes are indicated. (TIF) [file pone.0318142.s001.tif]

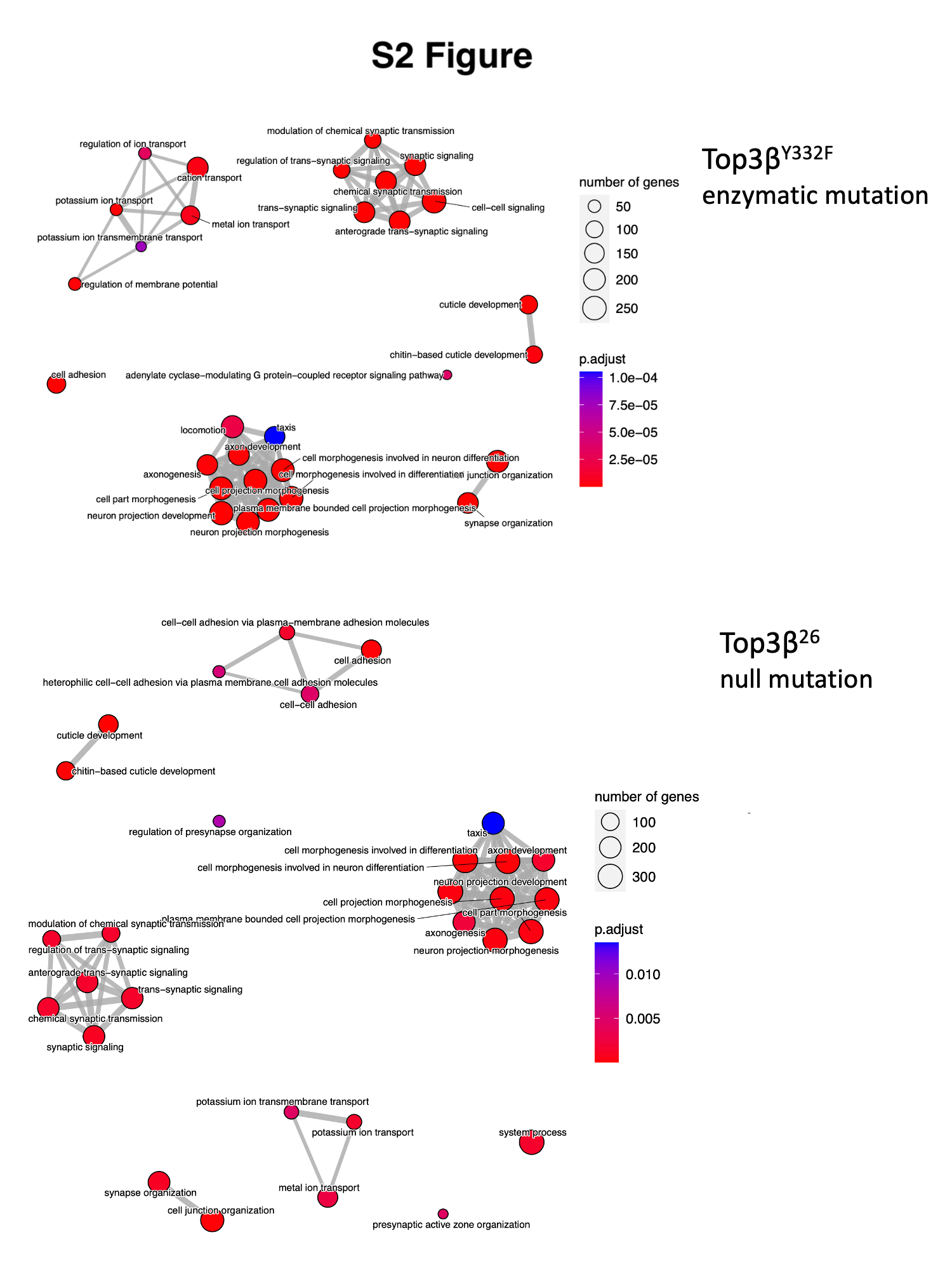

Supplement: S2 Fig — The enzymatic dead mutant Top3𝛽Y332F and the null mutant Top3𝛽26 show very similar effects on biological processes, indicating that the Tyr in the active site is crucial for the role of Topβ in the expression of normal mRNA levels for these Biological Processes. The Top3𝛽∆RGG mutant did not reveal enrichment terms. (TIF) [file pone.0318142.s002.tif]

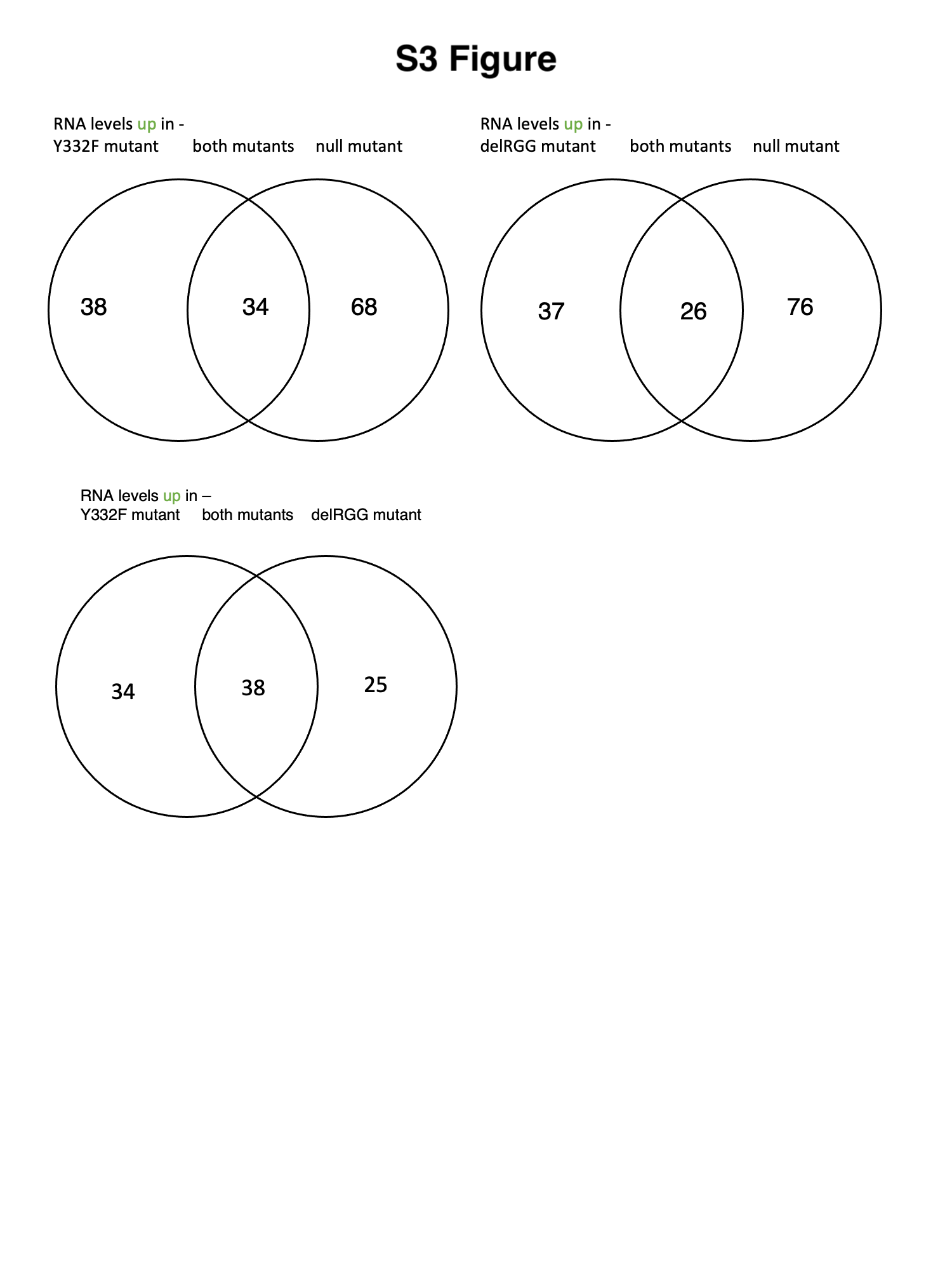

Supplement: S3 Fig — Genes showing higher transcript levels in 0–2 hrs old embryos mutant for Top3𝛽 (compared to their wild-type expression). Adip < 0.0002; log2 fold changes ≥ 1. The Venn diagram shows the pairwise overlap between the different mutants. (TIF) [file pone.0318142.s003.tif]

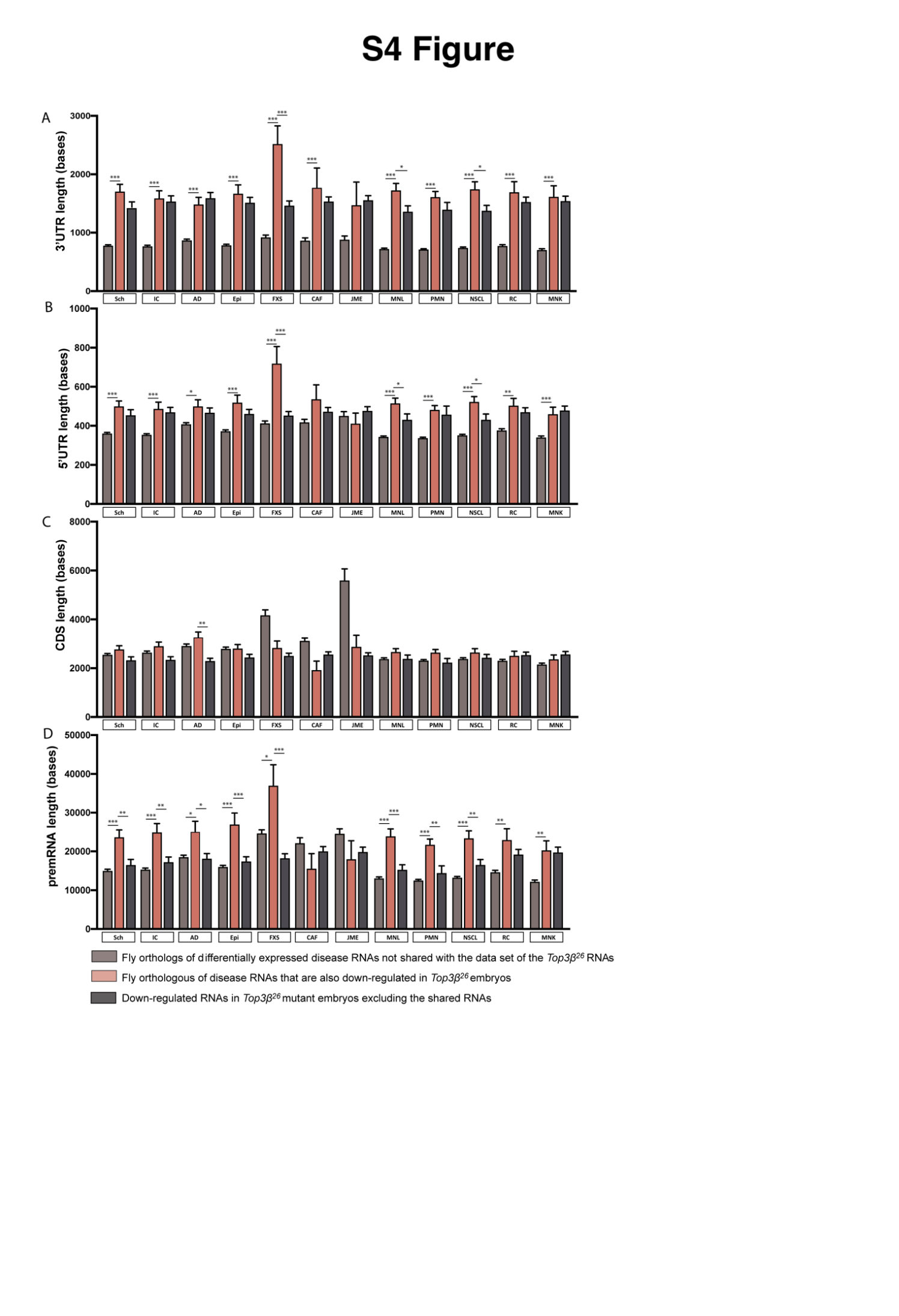

Supplement: S4 Fig — A, B) mRNAs shared between the two lists show generally longer UTRs compared to the fly orthologues of differentially expressed disease RNAs that are not downregulated in the Top3𝛽26 mutant embryos. C) CDS length revealed no significant difference between the three gene lists for each disease (except for AD). D) Shared mRNAs show generally longer pre-mRNAs. p-value < 0.0001=****, p-value < 0.001=***, p-value < 0.01=**, p-value < 0.05=*. Sch: Schizophrenia, IC: Impaired cognition, AD: Autistic Disorders, Epi: Epilepsy, FXS: Fragile X syndrome, CAF: Congenital anomaly of face, JME: Juvenile Myoclonic Epilepsy, MNL: Malignant neoplasm of the lung, PMN: Primary malignant neoplasm, NSCL: Non-Small Cell lung carcinoma, RC: Renal carcinoma, MNK: Malignant neoplasm of the kidney. (TIF) [file pone.0318142.s004.tif]

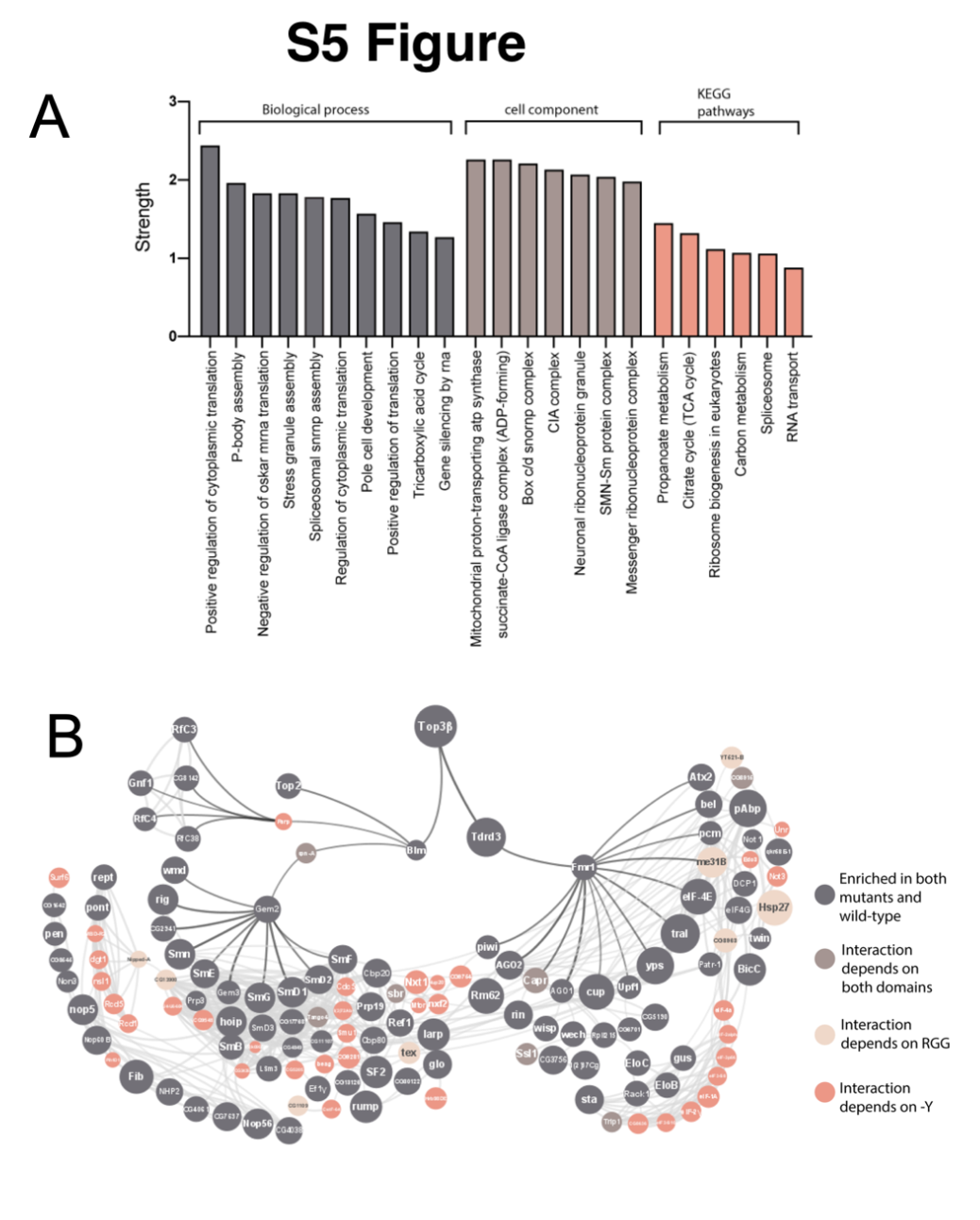

Supplement: S5 Fig — A) Gene ontology enrichment for Top3β-associated proteins for the top 100 interactors identified in embryonic extracts. Immunoprecipitations (IP) on extracts from 0–2 hours old embryos were performed using Top3𝛽::eGFP and the eGFP control line. Polypeptide components of the IP complexes were analyzed using mass spectrometry (MS), and their abundance was compared to the eGFP control. This resulted in a list of 426 potential complex components with an adjusted p-value < 0.01 and log2FC >1 (S9 Table). 89 ribosomal proteins were in this set. A gene ontology enrichment analysis was then performed for the top 100 (according to log2FC) non-ribosomal proteins. This revealed proteins involved in the activation of translation, P-bodies, stress granules, and neural ribonucleoprotein granules, all membrane-less structures involved in storing specific mRNAs during periods of stress or concentrating mRNAs and regulatory proteins [78]. Additionally, the interactors were also enriched in several functions related to mitochondria. B) Proteins are enriched in the embryonic protein-IP with their physical interactions, according to Cytoscape-String. Proteins that ended up further away from Top3β than the ones shown here, were removed from the interaction map. Tdrd3, an established interactor of Top3β [79], was among the top enriched proteins, suggesting that our immunoprecipitation was specific. To assess the role of the Y332 residue and the RGG box in Top3β interactions, IP results were compared with the ones from the Top3𝛽 mutants Top3𝛽Y332F::eGFP and Top3𝛽ΔRGG::eGFP. Among the 337 non-ribosomal binding candidates, 102 needed the Y residue and the RGG box to bind to Top3β::eGFP (S9 Table). An additional 16 proteins needed the Tyr (but not the RGG) and 23 the RGG box (but not the Y332) for their binding to Top3β. A large fraction of the identified proteins is involved in RNA transport, translation, splicing, mRNA surveillance, and degradation. The physical interaction map of the prote [file pone.0318142.s005.tif]

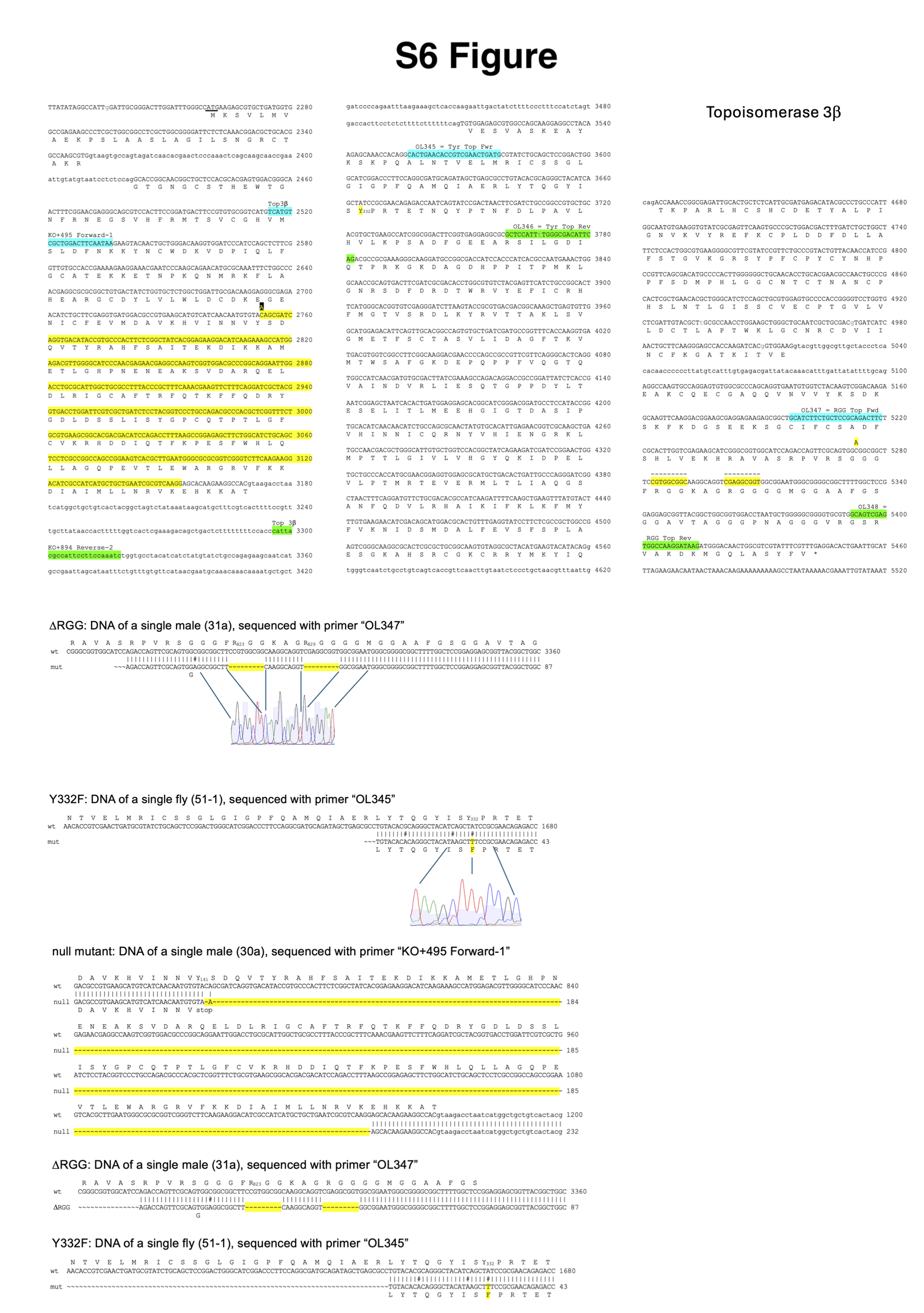

Supplement: S6 Fig — (TIF) [file pone.0318142.s006.tif]
