# Supplementary material for: Drosophila Topoisomerase 3β binds to mRNAs in vivo, contributes to their localization and stability, and counteracts premature aging
Source: PLoS One. 2025 Feb 11;20(2):e0318142. doi: 10.1371/journal.pone.0318142 (PMC12140118; doi:10.1371/journal.pone.0318142)
Supplement: Attachment — Submitted filename: response to reviewers.pdf [file pone.0318142.s022.pdf]

Reviewer #1: Top3b is a unique topoisomerase known for its ability to interact with and resolve complexities in both DNA and RNA. Mutations in Top3b have been linked to various psychological disorders, but how these mutations affect RNA processing and contribute to disease phenotypes is not well understood. Teimuri and Suter investigated the roles of Top3b in RNA metabolism, and found some evidence suggesting that deletion of Top3b and its catalytic mutant (Y332F) are associated with aging, developmental and neurological defects, potentially due to misregulation of target transcripts using Drosophila model. The results should be of interest to the topoisomerase field, and also add new knowledge on how Top3b mutation may affect neurodevelopment, neurodeneration, and aging. However, while their study uncovers some new aspects of Top3b's function in RNA metabolism, the authors need to perform some necessary experiments to improve the scientific quality of their data. The additional evidence is required to better support their conclusions and to meet standard of Plos One.

Major points

- The authors should provide concrete evidence to show that their newly made Top3b-mutant strains are correct. The authors should provide genomic DNA sequencing data for the mutant alleles.

==> We now provide more details in the Methods section, including at which steps the sequence of the mutant alleles was verified. We also deposited these strains at the stock center where they are freely available (the order numbers are listed in our Methods section). Because the mutagenesis method has become standard and the strains are freely available, we do not see a need to show the primary sequencing data in the paper but appended it to our response for the reviewer's perusal.

They also need to show Western Blotting for Top3b to confirm the null and normal levels of Top3b expression in Top3b26, Y332F-KI, and delta-RGG mutants, respectively.
=> The quantitative mass spectrometry (qMS) results presented in the Supplementary Table S5 are normalized to the eGFP-only expression done in parallel (and GFP is the target of the antibody). The log2 fold-change (compared to eGFP alone) is almost the same between Top3β^{+}::eGFP (4.7) and the mutant versions (4.6 for delRGG and 4.4 for Y332F) (lane 2 in the Supplementary Table S9). This result serves the same purpose as a Western blot. We now mention this result also in the Methods section. It is also consistent with the results of the embryonic stainings (Fig. 5) which were done with background controls and the same laser power and confocal microscope settings.
That *Top3b26* mRNA levels are down in the null mutant, we mention in the paper and refer to the quantification by RNAseq obtained by the analysis of its transcriptome (Supplementary Table S1; and S2-3 for the other mutants): Here are the values: for *Top3B*^{26}: 26/wt: 6.75 E50 (adjpv) / -2.32 (log2fc); for *Top3B*^{Y332F}: wt/Y332F: 0.33 (adjpv) / -0.19 (log2fc); for *Top3B* ^{deltaRGG}: wt/delRGG: 0.17 (adjpv) / -0.28 (log2fc). The "point" mutants do not show a reduction (but maybe a slightly higher mRNA expression level).

These experiments are critical controls to verify these Top3b-mutant strains.

- In Fig 1, the authors primarily utilized simple representations of their analysis lacking much information from their sequencing data. I suggest that the authors should incorporate more in-depth analysis techniques, such as volcano plots, dot distribution graphs, principal component analysis, etc. For instance, volcano plots for each mutant vs WT should be shown first so that readers can have an overall view (expression level and p-values) in the mutants. Then the venn graph can be followed. Add data points and mean values on all bar graphs.
==> We add the 3 volcano blots as Supplementary Figure S1 as a graphical representation of the Supplementary Tables S1-S3. They show the range of adj. p-values and the enrichment/reduction of the read numbers. We also add Venn diagrams for all comparisons with significant hits beause Reviewer 2 would like to see these (Fig. 1A,B; Fig. S3; Fig. 4). We also expanded the presentation of

the analysis by adding the RNAseq for genes (Fig. 1A; Fig. S1-S3) and explained more clearly, why we also analyzed RNAseq per transcript isoforms. Fig. S2 now shows the GO analysis in a different, more informative way. We changed the graphs into dot graphs with means as requested. This required changing the scale into log scale on the y-axis.

- For the RIP assay, the authors immunoprecipitated GFP-tagged Top3b using GFP antibodies. To validate the covalent binding of RNA to the -OH of the Y332 residue, they utilized GFP-Top3b-Y332F as a negative control. The problem of this assay is that GFP-Top3b-Y336F protein still has the RGG-box capable of binding to mRNAs non-covalently. Therefore, the authors need to do necessary controls to rule out whether the observed binding is co-valent or non-covalent. Can authors do some RIP-qPCR under both denaturing and non-denaturing conditions for some candidates to verify their observed difference between WT vs. Top3b-Y336F mutant?
=> We do not see the value of such an experiment. Binding to the RGG motif does not involve covalent interactions. These weaker interactions are generally not stable under our conditions with the ionic detergent SDS. Furthermore, unexpectedly strong interactions with the RGG motif would show the same binding to RGG in the mutant control and the wild type. It would, therefore, show up as a false negative and this is less of a problem than a false positive (because we found enough targets). Please also note that the very high number of different samples (different genotypes, different conditions, and all x3) forced us to focus on the most important controls, which we still think we did.

Moreover, they should include the RIP-seq assay for GFP-Top3b-RGG deletion mutant, which could allow them to identify mRNAs bound through RGG-box.
=> We performed these experiments as well but with different conditions (no SDS, no EDTA, no cross-linking, but with Mg^{++}). However, compared to the control without the two RGG motives, the wild-type IPs did not enrich specific mRNAs at a significant level. We, therefore, decided to focus fully on the binding to the active site. (BTW, relating to the previous discussion, this also suggests that the binding to the RGG motives is not very strong.)

Furthermore, they need to include an additional negative control that does not express GFP (e.g. ORR or w1118 background flies for their fly lines). This control would help rule out any non-specific binding of the GFP antibodies and the beads.
=> We do not agree with this. The chosen control Y>F weeds out all the background interactions that do not depend on the Y. Such non-specific binding happens with the Y>F::GFP as well and everything we obtain in the pulldown with material expressing GFP is background (BG). We do not need to know whether the background signal stems from the antibody, the beads, or from binding to GFP.

- To analyze the relationship between Top3b binding and RNA levels, the authors should conduct a comparative analysis of RIP-seq and RNA-seq data. This involves examining the overlap between the targets identified in RIP-seq, which represent genes bound by Top3b, and the genes showing differential expression in RNA-seq. This comparison will allow to determine if there is a direct effect of Top3b binding on RNA levels.
=> This was also our initial plan, but then we realized that the two experiments captured very different changes such that the comparison made little sense. We pointed out these differences already in the introduction and again in a slightly different context in the manuscript. We tried to improve this further in the new manuscript. The RNAseq gives us any difference in expression, regardless of whether it is a direct or indirect interaction. The RNAseq monitors also changes in chromatin structure (in the nurse cells of the ovary of the mother), transcription, splicing/RNA processing and nuclear export, and RNA stability, reflecting the effects of Top 3b on the RNAs and the DNA (because of its DNA topoisomerase activity). The RIPseq experiment gives us a quite different readout because it can only capture mature RNAs in the embryo itself. We described this already in the previous version but have now made a couple of changes and additions to this text.

“By capturing mRNA targets directly during the first 2 hours of embryogenesis, we should be able to focus on direct RNA targets of Top3β and avoid indirect effects. Furthermore, because this time window ends before zygotic transcription takes off, we do not expect Top3β to bind to pre-mRNAs in this assay and we should be able to focus on cytoplasmic interactions.”
Of course, we still compared the data sets and found too little overlap to present it. There are additional reasons why we might not get much overlap. We now present another one (although in a different context). The results of the analysis of the RNA <u>isoforms,</u> which show significant level reductions in the RNAseq, revealed that for a large fraction, there was another isoform that showed a just as significant reciprocal change. We now added this information. The changes are consistent with Top3B affecting transcription, splicing, and 3’end processing (which would happen at least mostly in the ovary) and would not lead to significant changes in RNAseq reads per gene. Also, we do not expect such changes to happen in the cytoplasm of the embryo.

- The authors discussed the possibility that Top3b may share the target transcripts with FMRP in discussion (Fig 7; they cited study done by Spradling group Science 2018). Top3b has been known to genetically and biochemically interact with Fmr1 (Xu 2013 NN). To investigate the functional and molecular roles of Top3b in collaboration with FMRP, it would be beneficial to compare its RNA targets with those of FMRP as the authors discussed. However, rather than referring to Science 2018, which is done in matured oocyte, I recommend utilizing data from Zhang et al. Nature Comms in 2022 (GEO dataset GSE143821). This dataset contains fine-scale timing RNA-seq data of Fmr1 mutant early embryos at different time points (0-0.5, 0.5-1, 1-2, 2-3 hours). By comparing the RNA targets of Top3b with those of Fmr1, they can assess any similarities or overlaps
=> We now used the datasets for the first 2 hours of development combined. This looks as follows:

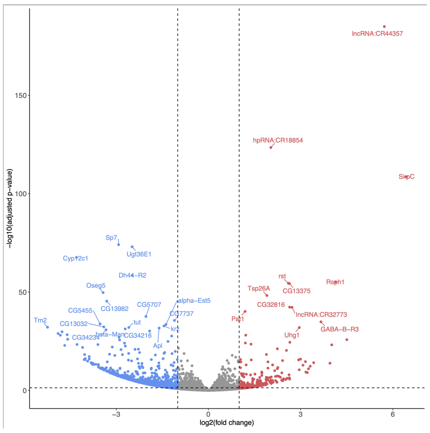

Comparing the “best” 30 hits from this blot with our top 289 differentially expressed isoforms targets in the null <u>and</u> Y332F mutants, we only found 1 (relatively weak hit), krz. Others may be high in one of them, but not the other (e.g. CG5455). It, therefore, does not seem to make much sense to incorporate this data. Additionally and as mentioned above, comparing bound targets in the embryo to RNAseq data where the RNA is produced in the mother is problematic. Instead, we compared the RNAseq from the embryonic FMR1 libraries with our RNAseq from the same embryonic stage and found the expected overlap of downregulated genes. This result is now in the new version as Fig. 4B and Supplementary Table S13.

- The authors stated that wild type Top3b and GFP tagged Top3b have similar expression level in Fig S5, but the supplementary materials do not have Fig S5 (has only Fig S1-4). The data may be in Table S5. A simple Western blotting with a proper loading control may be more accurate than mass spectrometry for this purpose.
=> Apologies for this typo. It should have been Table S5, not Supplementary Table S9. This is now corrected. Please note that the MS experiments were performed as quantitative MS (qMS), not simple MS. For a detailed discussion of this point, please see our second response on page 1.

Minor points

- On line 113-116, it is not clear how the authors determined the directly dependent on Top3b and obtained 315 RNAs. They need to clarify more details.

=> In the new version we changed this part and left out this probability-based estimate because it is less important and not worth lengthy explanations.

- In figure 2A, how reliable the association between two lists linked? Since the authors used embryos, I am not sure if the altered genes match with diseases linked genes. For example, does Fmr1 mutant embryo pick up many shared genes in AD or FXS? They need to validate if the method is reliable.

- In Figure 2A-B, the reliability of the association between the two lists of genes needs to be validated first. As the authors utilized embryos, it is unclear whether the altered genes identified in the study correspond to genes implicated in neuronal diseases. For instance, it would be essential to determine whether the genes identified in Fmr1 mutant embryos (see my major point above) overlap significantly with genes associated with AD or FXS.

==> We do not understand what the problem is and what this would tell us. I, therefore, try to explain a few different things that might be the reason for raising this point.

We only used genes that have already been implicated in these diseases based on their differential expression in the disease (of course there are way too many on the list to call them causative).

With the results presented in the present Suppl. Fig. S4, we were able to show that genes that are in the overlapping part of the Venn diagram show more extreme characteristics of Top3B targets than the ones outside the overlap (even the ones that are still Top3B-dependent). This should be a sign that the comparison has worked, too.

From what we know, FMR1 and Top3B do some things together but have also functions independent of the other. We also pointed out that these are different tissues (and organisms) and that despite this we obtained a good correlation. (One could expect that it would be even higher if the same tissue type had been used).

This does also not answer the question, butis still interesting in this context: half (16) of the FXS hits in Fig 2C (34) are also AD hits and more than half of the *FMR1* hits are also *Top3B26* hits (Figure 4B, Supplementary Table S13).

- In Fig 3, show datapoints and average values. Change the label ‘wild-type’ embryonic RNA-IP to ‘GFP-Top3b’ embryonic RNA-IP or do not use ‘wild-type’. It is no longer wild-type.

=> both are good points and the changes have been made.

- In Fig 3, the binding observed through the RIP appears relatively weak, as indicated by fold changes of less than 2(log2[2] = 4 fold). The authors should show qPCR validation of the RIP assay. For sequencing, I suggest utilizing DESEQ2 or other reliable programs to analyze the data and filter out non-significant p-values, thereby focusing on strong interactors.

=> We performed the RIPseq with three biological replicates and did not want to use the related technology to simply confirm the results obtained. Instead, we preferred to use this data to test candiadate RNAs for the biological significance of their interaction with Top3b, testing whether the encoded protein is being made at lower levels or is not localized properly. In this way, we did not only confirm a result but, simultanously, the work brought us a step further. We, therefore, see more value in the chosen approach. Our goal was to produce a list of enriched possible targets that are good candidate targets of Top3b. These good candidates can then be used to assess their biological significance of the individual interactions (for instance in the way we did it in Fig. 5). Please also note that in the topoisomerase cycle, the covalent bonds last only for a short fraction of the cycle. Even if we succeeded in stopping the reaction when we open the cells, only a small fraction of the possible target mRNAs will be covalently bound at this moment. (We now added this thought to the description of the experimental setup).

We used DESEQ2 with all RNAs we detected. The detected RNA targets were among the longer ones in A) and the ones that are significantly enriched (using DESEQ2) showed longer characteristics in the plots B, C, D, and E.

- Show or address the common characteristics of the homologous targets (length, isoform numbers, cellular localization, etc) if there are any in Fig 3F
=> Fig. 3F is now Fig. 4A and we added a table with these values.

- In Fig 4, The authors need to include counterstain for non-Top3b targets for the specificity of the changed targets. Also, some samples, particularly Kst, may exhibit high background. The cellular localization of Kst during early embryogenesis is strictly limited to apical membrane whereas the wildtype image shows apical & lateral regions with high intensity background in the non-membrane regions (Thomas and Kiehart Development 1994). Also, the authors should include quantification of the signals.
==> Because we needed to use the same settings for the wild-type and the mutant embryos, our apical stainings with anti-Kst in the wild-type embryos are overexposed and the weaker signal towards the center becomes visible. Weak signals can also be seen in the young embryos of the Thomas and Kiehart (1994) paper cited above (enlarged Fig. 3A,B and Fig. 4). Aside from differences in exposure and signal intensity, the contrast settings or antibody penetration in their Fig. 3 might also differ from ours. Among the different embryos in the stained samples, we always chose an example that showed an average staining. We feel that the points we make from this Figure (particularly the reduced localization in the absence of Top3b and its activity, but to some degree also the reduced protein levels) are supported by this Figure. To study in more detail individual proteins (which would be interesting), more work is required.

- For all images, the authors provide high mag images. All images are taken with low magnification and difficult to assess signals.
==> The reviewer would probably like to see higher mag pictures. We decided to show an intermediate magnification that shows the cortical (apical) localization and at the same time lateral, posterior, and central parts of the embryo in some detail. The qualitative differences between the wild type on the one side and the null and Y>F mutants are seen in the submitted Figure. The compressed pdf pictures are slightly less crisp than the ones we sent to the journal, but the staining differences that we describe should still be evident. However, we agree that a more detailed analysis of the individual defects would call for higher-power micrographs.

- It is interesting that Y332F exhibits strong nuclear localization. This observation raises the possibility that Y332F may manifest a gain-of-function phenotype rather than a loss-of-function. Alternatively, the observed phenotypes could arise from mis-localization of interacting proteins such as Tdrd3 and Fmrp, particularly if they form stable complexes and localized at the nucleus.
==> This is an interesting point, but the wild-type protein is nuclear, too! Only deltaRGG remains cytoplasmic. We also found that, unlike *deltaRGG, Y332F* behaves almost like the null mutant in probably all other assays. So what do the wild type and the *Y332F* mutant do in the nucleus if there is no transcription yet? Because Top3β is also a DNA topoisomerase and there are rapid mitotic cycles at this stage, I speculate that it might also help the other DNA topoisomerases (e.g. Top3α) to resolve topological DNA problems that arise during mitoses.

- For Y332F, address and include Top3bY332F staining in other large tissues (nurse cell or salivary gland) to confirm nuclear localization is general phenomenon.
==> We appreciate the interest and enthusiasm of this reviewer. There is so much interesting work that should be done on this fascinating topoisomerase. Unfortunately, we did not get to this

anymore, otherwise, we would include it in this reply. The manuscript is already loaded with data and we would not be able to study and cover it adequately in this manuscript. However, we hope that “someone” will be able to use these results and move on with this work after reading the paper.

- I do not see where the authors obtained Shot, Dhc and bH-spectrin(kst) antibodies. Cite the providers properly in the methods.
==> This is done now.

- Figure S4 showed that Top3b-KO and Y336F mutant strains display shortened lifespan. The data are consistent with earlier findings in mouse by James Wang and colleagues. The data should be of general interest and included in a main figure.
=> We did not even mention it anymore in this manuscript but forgot to remove Figure S4. Now we added it as Fig. 6C and mentioned this in the text. Now, we also mention the mouse result.

- The Discussion may be too long and can be shortened. The authors may want to reduce the redundancy with the Results.

=> we shortened the discussion, worked it over, and cut the redundancies.

Reviewer #2: Major conclusions of this manuscript are that Top3β and its catalytic activity are required to produce a considerable number of RNAs during drosophila embryonic development and to avoid deleterious neuromuscular phenotype.

These conclusion extends prior reports showing that: 1/ Particularly long pre-mRNAs and RNAs with long ORFs and long UTRs depend on Top3β for their normal expression levels, 2/ Top3β suppresses R-loops can therefore promote transcription, 2/ Top3β can promote splicing and the production of mature mRNA/ splicing isoforms, and 3/ Top3β is associated with RNA transport and help their proper localization in large cells, 5/ Top3β can stabilize a subset of RNAs, and 6/ Top3β can also promote translation of bound/ target RNAs. The authors should clearly discuss these possible mechanisms and cite pertinent references in relevant sections of the Introduction, Results and Discussion.
==> We added more references and discussions of mechanisms in these sections. We also point out how our results fit in with them, without overinterpretation and speculation.

The manuscript can be improved by addressing the following points:

1. Line 657-661: the same sentence is repeated twice.
==> This is now fixed

2. The way the author present data in figure 1A is confusing. The authors should show Venn diagram displaying overlap between downregulated genes in Top3β26, Top3βY332F, and Top3βΔRGG with respect to wild type. The overlapping downregulated RNAs between Top3β26 and Top3βY332F should ideally represent RNAs directly dependent on Top3β for their embryonic expression/ localization in embryo. Rest of the downregulated RNAs in Top3β26 embryo should represent RNAs that do not directly depend on Top3β catalytic activity.
==> We now present this additional data as requested and included volcano blots and more Venn diagrams. However, we cannot make the suggested “ideally” argument about genes being “directly dependent on Y332 (enzymatic activity). The RNAseq gives us any difference in expression,

regardless of whether it is a direct or indirect interaction. It monitors also changes in chromatin structure (in the nurse cells of the ovary of the mother), transcription, splicing/RNA processing and nuclear export, and RNA stability, reflecting the effects of Top3b on the RNAs and the DNA (because of its DNA topoisomerase activity). Compensatory effects or downstream effects show up as changes in the RNAseq, too. In the previous version, we tried to estimate what fraction is direct. We have now removed this part because it might have been a bit speculative and also because it is better to focus on the experimentally proven ones. In the new version, we now bring up the pairs of RNA isoforms that show reciprocal level changes. Some show differences in alternative splicing, changes in splice site selection, and in selecting the 3'end processing site. These changes would be consistent with a direct effect of Top3B's enzymatic activity.

3. Figures 1B-D: please mention number of RNAs in each of the groups mentioned here (either in results section or in figure/ figure legend).
==> We added the n= number in the legends for Fig 1C-E and the Figure for Fig. 3B-E.

4. Could the TOP3B CHIP seq be done in nurse cells/ maternal cells. Particularly, what is overlap between the RNAs downregulated in Top3β26, Top3βY332F embryos and genes that are bound specifically to wild-type Top3β? If Top3β bound genes that are overlapping with the RNAs downregulated in Top3β26, Top3βY332F embryos are also long, then only one can conclude that Top3β and its catalytic activity have a strong effect on the transcription of long mRNAs.
==> Assuming this question is about the RIPseq experiment, we have chosen the very young embryo before bulk transcription starts to avoid recording effects on transcription and splicing. Ideally, all RNAs are of maternal origin. The few zygotic ones that start coming up early and might be present at low numbers in our older stages are RNAs from Gap genes and genes involved in sex determination. These are all short genes and pre-mRNAs because only these manage to become transcribed during the short interphase of the syncytial cycles. Introducing the RIPseq experiments on p9-10, we point out why we expect the RNAseq and the RIPseq to give different datasets. In short, the RNAseq should record direct and indirect (e.g. compensatory or other downstream effects) differences that arise through the mutations. In the RNAseq we also monitor effects on chromatin structure, transcription, and splicing, but the RIPseq should primarily record cytoplasmic targets because there is no transcription at this stage (see also our reply at the bottom of page 2 and top of p3). Accordingly, we focused on exploring and explaining possible effects on RNA localization and translation and not on transcription and RNA processing. We have now elaborated a bit more on this on p9-10 to better explain it.

5. Downregulated RNAs in Top3β26, Top3βY332F embryos can also have problems with RNA maturation, RNA transport and RNA stability. What is the overlap between RNAs downregulated in Top3β26, Top3βY332F embryos and RNAs that are bound specifically to wild-type Top3β? If these overlapping RNAs display features like long ORFs, long UTRs then one can conclude that Top3β and its catalytic activity have a strong effect on the production of normal expression levels of long pre-mRNAs and RNAs with long ORFs and long UTRs in the embryos.
=> This was also our initial plan, but then we realized that the two experiments captured very different changes such that the comparison made little sense. I tried to explain this in a slightly different context in the manuscript and will elaborate on this here. The RNAseq gives us any difference in expression, regardless of whether it is a direct or indirect interaction. The RNAseq monitors also changes in chromatin structure (in the nurse cells of the ovary of the mother), transcription, splicing/RNA processing and nuclear export, and RNA stability, reflecting the effects of Top 3b on the RNAs and the DNA (because of its DNA topoisomerase activity). The RIPseq experiment gives us a quite different readout because it can only capture mature RNAs in the embryo itself. We described this already in the previous version but have now made a couple of changes and additions to this text. "By capturing mRNA targets directly during the first 2 hours of

embryogenesis, we should be able to focus on direct RNA targets of Top3β and avoid indirect effects. Furthermore, because this time window ends before zygotic transcription takes off, we do not expect Top3β to bind to pre-mRNAs in this assay and we should be able to focus on cytoplasmic interactions."
Of course, we still compared the data sets and found too little overlap to present it. There are additional reasons why we might not get much overlap. We now present another one (although in a different context). The results of the analysis of the RNA isoforms, which show significant level reductions in the RNAseq, revealed that for a large fraction, there was another isoform that showed a just as significant reciprocal change. We now added this information. The changes are consistent with Top3B affecting transcription, splicing, and 3'end processing (which would happen at least mostly in the ovary) and would not lead to significant changes in RNAseq reads per gene. Also, we do not expect such changes to happen in the cytoplasm of the embryo.

(Despite the differences in the experiments, the characteristics of the RNAs are very similar, suggesting that such RNAs run more frequently into problems and need TopB activity.)

6. The RNA immunoprecipitation is an important experiment for this study, yet the authors skipped over the issues of how pulled-down covalent TOP3B-RNA can be directly constructed into a sequencing library. A little more information on the generation of cDNA library will help the readers to better understand the scope and limitation of the method. Are random primers used?
==> We have now added this information: "For the library preparation, purified RNA was neither depleted for rRNA nor selected for poly(A+) RNA but used directly for reverse transcription with random primers".

7. The RNA immunoprecipitation was not carried out with the RGG-box deletion mutants, which would have provided some unique insights.
==> We did this in an analogous way but with $Mg2^{++}$ and without ionic detergent (and without crosslinking). When this RIPseq did not reveal significant targets, we decided to focus on our main interest, analyzing the enzymatic function toward RNA using the Y>F comparison.

8. Line 281: The author concluded that the Shot protein signal levels and localization were less affected in the RGG-deleted mutant embryos, but the images in Figure 4 do not support this conclusion. Please clarify.
==> Many thanks for pointing this out. I am not sure how this happened. Either it was a last-minute addition, thinking that we forgot to write it here, or I looked too much at the dots that seem to be slightly more abundant than in the other mutants. This part has been corrected (see also response under 13.)

9. In the results section "RNAs depending on Top3β for their normal expression" please define the nature of mutation in the Top3β mutant Top3β26 after you introduce it first time in the second line. It will benefit the readers.
==> This is now explained.

10. Results section "RNAs depending on Top3β for their normal expression", last 5 lines: the way authors argued and defined 315 RNAs as directly dependent on TOP3β for their expression is not very clear. Authors should clarify this statement.
=> Trying to explain it better by elaborating more on it, I decided to remove this crude estimation which depends on an assumption that could be seen as too speculative.

11. Supplementary Fig. 5 as mentioned in line 190 of result section "In vivo RNA interactions of Top3β in the absence of bulk transcription" not found within the submitted supplementary section. Please clarify.

==> Thank you for finding this typo, it should have been cited as Supplementary Table S5. This is now corrected and has become Supplementary Table S9.

12. Correlation between RNAs enriched in wild type Top3β IP compared to the Y332F mutant and their length (Fig. 3A) is not very strong (r=0.121). Please clarify "found that longer RNAs were indeed more likely to covalently bind to Top3β as they showed higher enrichment in the wild-type embryonic extracts (Fig. 3A)".
==> Please also note that in the topoisomerase cycle, the covalent bonds last only for a short time (fraction of the cycle). Even if we succeeded in stopping the reaction when we open the cells, only a small fraction of the possible target mRNAs will be covalently bound at this moment. (We now added this thought to the description of the experimental setup). Because of this, we can only capture a small fraction of a possible target and all the other RNAs from this gene will contribute to the background. We therefore knew from the start that we would be working close to the background and I think that the r-value and the enrichments we get reflect this. This is clearly different from standard co-IPs.

13. Figure 4: why Shot protein expression is weaker in Top3βΔRGG mutant even though shot mRNA expression remains unaltered with respect to wild type embryo.
==> (Maybe the RNA we see in the deltaRGG embryos cannot be translated because it has structural problems without becoming degraded). We now point out that this might reflect a role of Top3 β in translation and that this is consistent with previous results that demonstrated that Top3β is required for efficient translation of mRNAs (Ref 74).

14. Authors should also find out the RNAs pulled down by Top3β-ΔRGG protein. This can clarify the function of RGG domain in Top3B catalytic activity.
==> We did this in an analogous way but with $Mg2^{++}$ and without ionic detergent (and without crosslinking). When this RIPseq did not reveal significant targets, we decided to focus on our main interest, analyzing the enzymatic function toward RNA using the Y>F comparison.

Topoisomerase 3β

```
TTATATAGGCCATTgGATTGCGGGACTTGGATTTGGGCCATGAAGAGCGTGCTGATGGTG 2280
                                        M  K  S  V  L  M  V

GCCGAGAAGCCCTCGCTGGCGGCCTCGCTGGCGGGGATTCTCTCAAACGGACGCTGCACG 2340
 A  E  K  P  S  L  A  A  S  L  A  G  I  L  S  N  G  R  C  T

GCCAAGCGTGgtaagtgccagtagatcaacacgaactcccaaactcagcaagcaaccgaa 2400
 A  K  R

attgtatgtaatcctctccagGCACCGGCAACGGCTGCTCCACGCACGAGTGGACGGGCA 2460
                      G  T  G  N  G  C  S  T  H  E  W  T  G

                                                       Top3β
ACTTTCGGAACGAGGGCAGCGTCCACTTCCGGATGACTTCCGTGTGCGGTCATGTCATGT 2520
N  F  R  N  E  G  S  V  H  F  R  M  T  S  V  C  G  H  V  M

KO+495 Forward-1
CGCTGGACTTCAATAAGAAGTACAACTGCTGGGACAAGGTGGATCCCATCCAGCTCTTCG 2580
S  L  D  F  N  K  K  Y  N  C  W  D  K  V  D  P  I  Q  L  F

GTTGTGCCACCGAAAAGAAGGAAACGAATCCCAAGCAGAACATGCGCAAATTTCTGGCCC 2640
G  C  A  T  E  K  K  E  T  N  P  K  Q  N  M  R  K  F  L  A

ACGAGGCGCGCGGCTGTGACTATCTGGTGCTCTGGCTGGATTGCGACAAGGAGGGCGAGA 2700
H  E  A  R  G  C  D  Y  L  V  L  W  L  D  C  D  K  E  G  E
                                                    A
ACATCTGCTTCGAGGTGATGGACGCCGTGAAGCATGTCATCAACAATGTGTACAGCGATC 2760
N  I  C  F  E  V  M  D  A  V  K  H  V  I  N  N  V  Y  S  D

AGGTGACATACCGTGCCCACTTCTCGGCTATCACGGAGAAGGACATCAAGAAAGCCATGG 2820
Q  V  T  Y  R  A  H  F  S  A  I  T  E  K  D  I  K  K  A  M

AGACGTTGGGGCATCCCAACGAGAACGAGGCCAAGTCGGTGGACGCCCGGCAGGAATTGG 2880
E  T  L  G  H  P  N  E  N  E  A  K  S  V  D  A  R  Q  E  L

ACCTGCGCATTGGCTGCGCCTTTACCCGCTTTCAAACGAAGTTCTTTCAGGATCGCTACG 2940
D  L  R  I  G  C  A  F  T  R  F  Q  T  K  F  F  Q  D  R  Y

GTGACCTGGATTCGTCGCTGATCTCCTACGGTCCCTGCCAGACGCCCACGCTCGGTTTCT 3000
G  D  L  D  S  S  L  I  S  Y  G  P  C  Q  T  P  T  L  G  F

GCGTGAAGCGGCACGACGACATCCAGACCTTTAAGCCGGAGAGCTTCTGGCATCTGCAGC 3060
C  V  K  R  H  D  D  I  Q  T  F  K  P  E  S  F  W  H  L  Q

TCCTCGCCGGCCAGCCGGAAGTCACGCTTGAATGGGCGCGCGGTCGGGTCTTCAAGAAGG 3120
L  L  A  G  Q  P  E  V  T  L  E  W  A  R  G  R  V  F  K  K

ACATCGCCATCATGCTGCTGAATCGCGTCAAGGAGCACAAGAAGGCCACgtaagacctaa 3180
D  I  A  I  M  L  L  N  R  V  K  E  H  K  K  A  T

tcatggctgctgtcactacggctagtctataaataagcatgctttcgtcacttttccgtt 3240

                                                       Top 3β
tgcttataaccactttttggtcactcgaaagacagctgactctttttttttccacccatta 3300

KO+894 Reverse-2
cgccattccttccaaatctggtgcctacatcatctatgtatctgccagagaagcaatcat 3360

gccgaattagcataatttctgtttgtgttcataacgaatgcaaacaaacaaaatgctgct 3420

gatccccagaatttaagaaagctcaccaagaattgactatcttttccctttccatctagt 3480

gaccacttcctctcttttctttttttcagTGTGGAGAGCGTGGCCAGCAAGGAGGCCTACA 3540
                              V  E  S  V  A  S  K  E  A  Y

                 OL345 = Tyr Top Fwr
AGAGCAAACCACAGGCACTGAACACCGTCGAACTGATGCGTATCTGCAGCTCCGGACTGG 3600
K  S  K  P  Q  A  L  N  T  V  E  L  M  R  I  C  S  S  G  L

GCATCGGACCCTTCCAGGCGATGCAGATAGCTGAGCGCCTGTACACGCAGGGCTACATCA 3660
G  I  G  P  F  Q  A  M  Q  I  A  E  R  L  Y  T  Q  G  Y  I

GCTATCCGCGAACAGAGACCAATCAGTATCCGACTAACTTCGATCTGCCGGCCGTGCTGC 3720
S  Y332P  R  T  E  T  N  Q  Y  P  T  N  F  D  L  P  A  V  L

                                           OL346 = Tyr Top Rev
ACGTGCTGAAGCCATCGGCGGACTTCGGTGAGGAGGCGCGCTCCATTtTGGGCGACATTC 3780
H  V  L  K  P  S  A  D  F  G  E  E  A  R  S  I  L  G  D  I

AGACGCCGCGAAAGGGCAAGGATGCCGGCGACCATCCACCCATCACGCCAATGAAACTGG 3840
Q  T  P  R  K  G  K  D  A  G  D  H  P  P  I  T  P  M  K  L

GCAACCGCAGTGACTTCGATCGCGACACCTGGCGTGTCTACGAGTTCATCTGCCGGCACT 3900
G  N  R  S  D  F  D  R  D  T  W  R  V  Y  E  F  I  C  R  H

TCATGGGCACGGTGTCGAGGGATCTTAAGTACCGCGTGACGACGGCAAAGCTGAGTGTTG 3960
F  M  G  T  V  S  R  D  L  K  Y  R  V  T  T  A  K  L  S  V

GCATGGAGACATTCAGTTGCACGGCCAGTGTGCTGATCGATGCCGGTTTCACCAAGGTGA 4020
G  M  E  T  F  S  C  T  A  S  V  L  I  D  A  G  F  T  K  V

TGACGTGGTCGGCCTTCGGCAAGGACGAACCCCAGCCGCCGTTCGTTCAGGGCACTCAGG 4080
M  T  W  S  A  F  G  K  D  E  P  Q  P  P  F  V  Q  G  T  Q

TGGCCATCAACGATGTGCGACTTATCGAAAGCCAGACAGGACCGCCGGATTATCTCACCG 4140
V  A  I  N  D  V  R  L  I  E  S  Q  T  G  P  P  D  Y  L  T

AATCGGAGCTAATCACACTGATGGAGGAGCACGGCATCGGGACGGATGCCTCCATACCGG 4200
E  S  E  L  I  T  L  M  E  E  H  G  I  G  T  D  A  S  I  P

TGCACATCAACAACATCTGCCAGCGCAACTATGTGCACATTGAGAACGGTCGCAAGCTGA 4260
V  H  I  N  N  I  C  Q  R  N  Y  V  H  I  E  N  G  R  K  L

TGCCAACGACGCTGGGCATTGTGCTGGTCCACGGCTATCAGAAGATCGATCCGGAACTGG 4320
M  P  T  T  L  G  I  V  L  V  H  G  Y  Q  K  I  D  P  E  L

TGCTGCCCACCATGCGAACGGAGGTGGAGCGCATGCTGACACTGATTGCCCAGGGATCGG 4380
V  L  P  T  M  R  T  E  V  E  R  M  L  T  L  I  A  Q  G  S

CTAACTTTCAGGATGTTCTGCGACACGCCATCAAGATTTTCAAGCTGAAGTTTATGTACT 4440
A  N  F  Q  D  V  L  R  H  A  I  K  I  F  K  L  K  F  M  Y

TTGTGAAGAACATCGACAGCATGGACGCACTGTTTGAGGTATCCTTCTCGCCGCTGGCCG 4500
F  V  K  N  I  D  S  M  D  A  L  F  E  V  S  F  S  P  L  A

AGTCGGGCAAGGCGCACTCGCGCTGCGGCAAGTGTAGGCGCTACATGAAGTACATACAGg 4560
E  S  G  K  A  H  S  R  C  G  K  C  R  R  Y  M  K  Y  I  Q

tgggtcaatctgcctgtcagtcaccgttcaacttgtaatctccctgctaacgtttaattg 4620

cagACCAAACCGGCGAGATTGCACTGCTCTCATTGCGATGAGACATACGCCCTGCCCATT 4680
   T  K  P  A  R  L  H  C  S  H  C  D  E  T  Y  A  L  P  I

GGCAATGTGAAGGTGTATCGCGAGTTCAAGTGCCCGCTGGACGACTTTGATCTGCTGGCT 4740
 G  N  V  K  V  Y  R  E  F  K  C  P  L  D  D  F  D  L  L  A

TTCTCCACTGGCGTGAAGGGGCGTTCGTATCCGTTCTGCCCGTACTGTTACAACCATCCG 4800
 F  S  T  G  V  K  G  R  S  Y  P  F  C  P  Y  C  Y  N  H  P

CCGTTCAGCGACATGCCCCACTTGGGGGGCTGCAACACCTGCACGAACGCCAACTGCCCG 4860
 P  F  S  D  M  P  H  L  G  G  C  N  T  C  T  N  A  N  C  P

CACTCGCTGAACACGCTGGGCATCTCCAGCTGCGTGGAGTGCCCCACCGGGGTCCTGGTG 4920
 H  S  L  N  T  L  G  I  S  S  C  V  E  C  P  T  G  V  L  V

CTCGATTGTACGCTTtGCGCCAACCTGGAAGCTGGGCTGCAATCGCTGCGACgTGATCATC 4980
 L  D  C  T  L  A  P  T  W  K  L  G  C  N  R  C  D  V  I  I

AACTGCTTCAAGGGAGCCACCAAGATCACgGTGGAAGgtacgttggcgttgctaccctca 5040
 N  C  F  K  G  A  T  K  I  T  V  E

cacaaccccccttatgtcatttgtgagacgattatacaaacatttgattatattttgcag 5100

AGGCCAAGTGCCAGGAGTGTGGCGCCCAGCAGGTGAATGTGGTCTACAAGTCGGACAAGA 5160
E  A  K  C  Q  E  C  G  A  Q  Q  V  N  V  V  Y  K  S  D  K

                                          OL347 = RGG Top Fwd
GCAAGTTCAAGGACGGAAGCGAGGAGAAGAGCGGCTGCATCTTCTGCTCCGCAGACTTCT 5220
S  K  F  K  D  G  S  E  E  K  S  G  C  I  F  C  S  A  D  F

                                                        A
CGCACTTGGTCGAGAAGCATCGGGCGGTGGCATCCAGACCAGTTCGCAGTGGCGGCGGCT 5280
S  H  L  V  E  K  H  R  A  V  A  S  R  P  V  R  S  G  G  G

  ---------        ---------
TCCGTGGCGGCAAGGCAGGTCGAGGCGGTGGCGGAATGGGCGGGGCGGCTTTTGGCTCCG 5340
F  R  G  G  K  A  G  R  G  G  G  G  M  G  G  A  A  F  G  S

                                                       OL348 =
GAGGAGCGGTTACGGCTGGCGGTGGACCTAATGCTGGGGGCGGGGTGCGTGGCAGTCGAG 5400
G  G  A  V  T  A  G  G  G  P  N  A  G  G  G  V  R  G  S  R

 RGG Top Rev
TGGCCAAGGATAAGATGGGACAACTGGCGTCGTATTTCGTTTGAGGACACTGAATTGCAT 5460
V  A  K  D  K  M  G  Q  L  A  S  Y  F  V  *

TTAGAAGAACAATAACTAAACAAGAAAAAAAAAGCCTAATAAAAACGAATTGTATAAAT 5520
```

ΔRGG: DNA of a single male (31a), sequenced with primer “OL347”

```
      R  A  V  A  S  R  P  V  R  S  G  G  G  F R823 G  G  K  A  G R829 G  G  G  M  G  G  A  A  F  G  S  G  G  A  V  T  A  G
wt   CGGGCGGTGGCATCCAGACCAGTTCGCAGTGGCGGCGGCTTCCGTGGCGGCAAGGCAGGTCGAGGCGGTGGCGGAATGGGCGGGGCGGCTTTTGGCTCCGGAGGAGCGGTTACGGCTGGC 3360
                    |||||||||||||||||#||||||||         ||||||||||         |||||||||||||||||||||||||||||||||||||||||||||||||||
mut             ~~~AGACCAGTTCGCAGTGGAGGCGGCTT---------CAAGGCAGGT---------GGCGGAATGGGCGGGGCGGCTTTTGGCTCCGGAGGAGCGGTTACGGCTGGC 87
                                     G
```

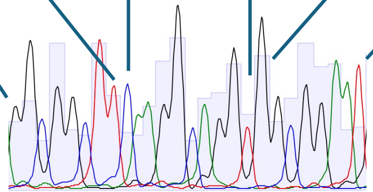

Y332F: DNA of a single fly (51-1), sequenced with primer “OL345”

```
      N  T  V  E  L  M  R  I  C  S  S  G  L  G  I  G  P  F  Q  A  M  Q  I  A  E  R  L  Y  T  Q  G  Y  I  S Y332 P  R  T  E  T
wt   AACACCGTCGAACTGATGCGTATCTGCAGCTCCGGACTGGGCATCGGACCCTTCCAGGCGATGCAGATAGCTGAGCGCCTGTACACGCAGGGCTACATCAGCTATCCGCGAACAGAGACC 1680
                                                                                    |||||||#|||||||||||#||||#||||||||||||||||
mut                                                                              ~~~TGTACACACAGGGCTACATAAGCTTTCCGCGAACAGAGACC 43
                                                                                   L  Y  T  Q  G  Y  I  S  F  P  R  T  E  T
```

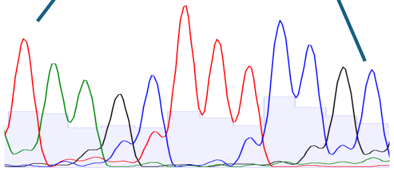

null mutant: DNA of a single male (30a), sequenced with primer “KO+495 Forward-1”

```
       D  A  V  K  H  V  I  N  N  V Y141 S  D  Q  V  T  Y  R  A  H  F  S  A  I  T  E  K  D  I  K  K  A  M  E  T  L  G  H  P  N
wt    GACGCCGTGAAGCATGTCATCAACAATGTGTACAGCGATCAGGTGACATACCGTGCCCACTTCTCGGCTATCACGGAGAAGGACATCAAGAAAGCCATGGAGACGTTGGGGCATCCCAAC 840
      |||||||||||||||||||||||||||||||| |
null  GACGCCGTGAAGCATGTCATCAACAATGTGTA-A-------------------------------------------------------------------------------------- 184
       D  A  V  K  H  V  I  N  N  V stop

       E  N  E  A  K  S  V  D  A  R  Q  E  L  D  L  R  I  G  C  A  F  T  R  F  Q  T  K  F  F  Q  D  R  Y  G  D  L  D  S  S  L
wt    GAGAACGAGGCCAAGTCGGTGGACGCCCGGCAGGAATTGGACCTGCGCATTGGCTGCGCCTTTACCCGCTTTCAAACGAAGTTCTTTCAGGATCGCTACGGTGACCTGGATTCGTCGCTG 960

null  ------------------------------------------------------------------------------------------------------------------------ 185

       I  S  Y  G  P  C  Q  T  P  T  L  G  F  C  V  K  R  H  D  D  I  Q  T  F  K  P  E  S  F  W  H  L  Q  L  L  A  G  Q  P  E
wt    ATCTCCTACGGTCCCTGCCAGACGCCCACGCTCGGTTTCTGCGTGAAGCGGCACGACGACATCCAGACCTTTAAGCCGGAGAGCTTCTGGCATCTGCAGCTCCTCGCCGGCCAGCCGGAA 1080

null  ------------------------------------------------------------------------------------------------------------------------ 185

       V  T  L  E  W  A  R  G  R  V  F  K  K  D  I  A  I  M  L  L  N  R  V  K  E  H  K  K  A  T
wt    GTCACGCTTGAATGGGCGCGCGGTCGGGTCTTCAAGAAGGACATCGCCATCATGCTGCTGAATCGCGTCAAGGAGCACAAGAAGGCCACgtaagacctaatcatggctgctgtcactacg 1200
                                                                               |||||||||||||||||||||||||||||||||||||||||||||||
null  -------------------------------------------------------------------------AGCACAAGAAGGCCACgtaagacctaatcatggctgctgtcactacg 232
```

ΔRGG: DNA of a single male (31a), sequenced with primer “OL347”

```
        R  A  V  A  S  R  P  V  R  S  G  G  G  F R823 G  G  K  A  G  R  G  G  G  G  M  G  G  A  A  F  G  S
wt      CGGGCGGTGGCATCCAGACCAGTTCGCAGTGGCGGCGGCTTCCGTGGCGGCAAGGCAGGTCGAGGCGGTGGCGGAATGGGCGGGGCGGCTTTTGGCTCCGGAGGAGCGGTTACGGCTGGC 3360
                       |||||||||||||||||#||||||||         ||||||||||         |||||||||||||||||||||||||||||||||||||||||||||||||||
ΔRGG    ~~~~~~~~~~~~~~~AGACCAGTTCGCAGTGGAGGCGGCTT---------CAAGGCAGGT---------GGCGGAATGGGCGGGGCGGCTTTTGGCTCCGGAGGAGCGGTTACGGCTGGC 87
                                        G
```

Y332F: DNA of a single fly (51-1), sequenced with primer “OL345”

```
     N  T  V  E  L  M  R  I  C  S  S  G  L  G  I  G  P  F  Q  A  M  Q  I  A  E  R  L  Y  T  Q  G  Y  I  S Y332 P  R  T  E  T
wt   AACACCGTCGAACTGATGCGTATCTGCAGCTCCGGACTGGGCATCGGACCCTTCCAGGCGATGCAGATAGCTGAGCGCCTGTACACGCAGGGCTACATCAGCTATCCGCGAACAGAGACC 1680
                                                                                    |||||||#|||||||||||#||||#||||||||||||||||
mut  ~~~~~~~~~~~~~~~~~~~~~~~~~~~~~~~~~~~~~~~~~~~~~~~~~~~~~~~~~~~~~~~~~~~~~~~~~~~~~~~TGTACACACAGGGCTACATAAGCTTTCCGCGAACAGAGACC 43
                                                                                    L  Y  T  Q  G  Y  I  S  F  P  R  T  E  T
```
