# Supplementary material for: Drosophila Topoisomerase 3β binds to mRNAs in vivo, contributes to their localization and stability, and counteracts premature aging
Source: PLoS One. 2025 Feb 11;20(2):e0318142. doi: 10.1371/journal.pone.0318142 (PMC12140118; doi:10.1371/journal.pone.0318142)
Supplement: Attachment — Submitted filename: Response to Second Review Nov 2024.pdf [file pone.0318142.s023.pdf]

6. Review Comments to the Author

Please use the space provided to explain your answers to the questions above. You may also include additional comments for the author, including concerns about dual publication, research ethics, or publication ethics. (Please upload your review as an attachment if it exceeds 20,000 characters)

Reviewer #1: In this version, the authors addressed some but not all of my requests in my previous review. The authors often claim that they do not agree with my specific comments and requests. As a result, the manuscript has limited improvement, and its major problems still remain. I therefore cannot recommend its publication.

➔ I appreciate the constructive comments this reviewer gave us and we made changes to improve the manuscript. However, we refrained from performing experiments that would not add new value to the paper. I tried to explain our arguments already in the previous response file (added again in the detailed part below in green).

Here are just some additional comments.

1. The authors did provide the sequencing data for their newly-generated Top3b mutant strains in their response letter. To me, these data are important to confirm that the new strains are correct. In my opinion, the data should be included in a Supplemental Figure.

➔ We have no problem doing this if the editor and journal request it (please consider that we already have numerous suppl. Figs. & Tables). I have now included this, but depending on the editorial decision, I could remove it again. Please let me know.
Our previous response was:
"==> We now provide more details in the Methods section, including at which steps the sequence of the mutant alleles was verified. We also deposited these strains at the stock center, where they are freely available (the order numbers are listed in our Methods section). Because the mutagenesis method has become standard and the strains are freely available, we do not see a need to show the primary sequencing data in the paper but appended it to our response for the reviewer's perusal."

2. To address my question whether the Top3b mutant proteins are expressed at similar levels to that of WT in newly-generated strains, the authors provided IP-Mass data for GFP-Top3b-WT and different mutant proteins. But these data are not what I am looking for. My question is whether endogenous Top3b-WT, Y336F and dRGG proteins are expressed at similar levels. For example, if the mutant proteins are expressed at lower levels than that of WT, one can argue that the observed phenotype in the mutant flies is due to reduced mutant protein, rather than due to loss of Top3b catalytic or RNA binding activity. The authors should examine the endogenous Top3b-WT and mutant protein levels in WT, KO, vs. Y336F, vs. dRGG flies by Western blotting. The authors claimed that they have RNA-seq data showing that Top3b RNA level is reduced in Top3b-KO, but not Y336F or dRGG mutants. But RNA levels do not equal to protein levels.

➔ I fully understand the concern raised here. However, we have already shown in different ways that this is not the case. First of all, the reviewer's concern applies mainly the Y332F mutant, the focus of this manuscript.
- From the stainings of the mutant embryos, where the same settings were used for the null mutant, the wild type, and the point mutants (Fig. 5, last column), it appears that the Y322F protein is expressed at levels indistinguishable from the ones of the wild-type protein.
- In this context, the fact that the *Top3β* RNA levels are down in the $Top3\beta^{26}$ null mutant but not in the two point mutants (Supplementary Table S1-3) not only shows that the mutant RNA is expressed at the same level as the wild-type RNA, it is also consistent with the

above interpretation of the Figure 5 results, which shows normal Top3β protein staining levels for the point mutants but no (or background) signal for Top3β^{26}.
- We now know that the RNA is made at the same level, that it is translated and produces a stable protein. With this knowledge, we maintain that we can address the quantitative aspects of the protein expression and stability using the quantitative MS (qMS) assay with the GFP-tagged protein (Supplementary Table S9). In the criticism, Reviewer 1 still insists on a Western blot instead of "MS" (in the second review, the reviewer mentioned only "MS", overlooking or ignoring the fact that it was quantitative MS). Quantitive MS analysis, done properly, is at least as good as Western blotting.

(For the delRGG mutant, which is not the focus of this manuscript, we have the following arguments:
- Genetically, it behaves way more like the wild type at the stages we examined. It cannot be a null or close to a null mutant.
- RNA levels are also normal
- qMS data shows that the tagged protein is expressed normally
- protein staining of embryos shows reduced staining in the nuclei, but not in the cytoplasm where the reported interactions take place (there is virtually no transcription at this stage)).

Here is what we responded previously:
=> The quantitative mass spectrometry (qMS) results presented in the Supplementary Table S5 are normalized to the eGFP-only expression done in parallel (and GFP is the target of the antibody). The log2 fold-change (compared to eGFP alone) is almost the same between Top3β^{+}::eGFP (4.7) and the mutant versions (4.6 for delRGG and 4.4 for Y332F) (lane 2 in the Supplementary Table S9). This result serves the same purpose as a Western blot. We now mention this result also in the Methods section. It is also consistent with the results of the embryonic stainings (Fig. 5) which were done with background controls and the same laser power and confocal microscope settings.
That *Top3b26* mRNA levels are down in the null mutant, we mention in the paper and refer to the quantification by RNAseq obtained by the analysis of its transcriptome (Supplementary Table S1; and S2-3 for the other mutants): Here are the values: for *Top3B*^{26}: 26/wt: 6.75 E50 (adjpv) / -2.32 (log2fc); for *Top3B*^{Y332F}: wt/Y332F: 0.33 (adjpv) / -0.19 (log2fc); for *Top3B* ^{deltaRGG}: wt/delRGG: 0.17 (adjpv) / -0.28 (log2fc). The "point" mutants do not show a reduction (but maybe a slightly higher mRNA expression level).

3. The authors have certainly improved the RNA-seq data presentation with the addition of new volcano plots and dot graphs, which provide a clearer and more comprehensive visualization of the data. However, I still have concerns regarding the data tables and their consistency with the figures. Specifically, there are discrepancies between the results presented in the figures and those in the supplementary tables. For example, in analyzing Table S1, there are 2,656 downregulated and 272 upregulated genes (log2>1, Adjp<0.05), whereas Fig 1a shows a much higher number of differentially expressed genes (DEGs). Similarly, Tables S2 and S3 show inconsistencies with the figures, which suggests that less stringent filtering may have been applied. This could be also due to annotation issues as the Supp tables contains protein coding genes, pseudogenes, transposons and other non-coding RNAs, and I believe the authors' main focus is protein coding mRNAs. The authors should double-check their analysis and correct any discrepancies in the reported numbers.

➔ I appreciate that this issue was pointed out. The discrepancy was due to using rounded (log2fc) values and log2fc ≥1 in one case and the more precise log2fc >1 in the other. In the new version, we now use log2fc >1 for both (without arbitrary rounding). Additionally, because I see less value in using DEGs, I reanalyzed the data and presented down- and upregulated genes separately in Fig 1A using <-1 and >1 values instead of ≤ and ≥.
This is the new version of Fig. 1A:

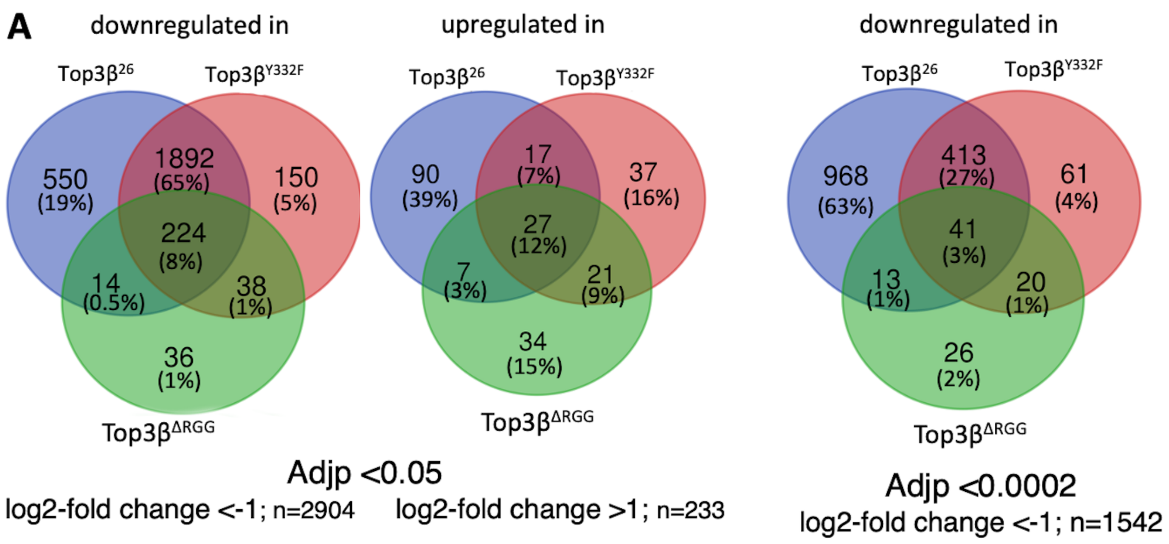

4. The authors did not perform any experiments to rule out that the binding is due to covalent or non-covalent. Without such controls, I am not convinced their reported binding is due to the covalent cleavage complex, or non-covalent through RGG or other nucleic acid-binding motifs. I agree that GFP-Top3b-Y332F may be a control to reduce non-covalently bound RNAs. However, due to the relatively low fold enrichment values observed in the RIP-seq data (e.g., the strongest interaction show ~3-fold enrichment in Table S10, which is somewhat low for RNA-IP experiments), I still think that it is necessary to perform RIP-qPCR support their RIP-seq data and to validate conclusions of their study.

➔ I tried to explain why this request does not make sense to me. The only difference between the protein that can bind the RNA covalently and the control is the one -OH group that makes to covalent bond. Of course, RNAs are known to bind to the RGG motives (and possibly other regions, too), but these are non-covalent interactions that will be washed off by SDS. I have added the following sentence to the description of the method to bring this point across: "…because RNAs non-covalently interacting with Top3β will be washed off the enzyme by the ionic detergent." Of course, there will still be background levels of mRNAs that non-covalently stick to the protein, but these will be present in the control, too, and at the same "background level" in the IP with the functional enzyme and the enzymatically dead one. Therefore, they will be identified as non-specific backgrounds.

We expected that the true enrichment cannot be very high because the covalent phase is only part of the enzymatic cycle and only a fraction of an RNA species interacts with the enzyme at a given time (we pointed this out already, but elaborated a bit more on this on p12/13). Please note that this is much different from an RNA-binding protein that sits continuously on an mRNA because only a small fraction of a specific RNA pool is bound to Top3B at one time. (On the other hand, theoretically, every expressed mRNA could become a Top3B target.)

Furthermore, RIP-qPCR relies on the same sample preparation as the RIP-seq experiments that we had performed in biological triplicates. Also, variations of the RIP protocol (e.g., artificial cross-linking) would prevent comparison with our presented dataset. Trying to use our resources efficiently, it seemed to make much more sense to further test the value of the results by testing for the predicted effects on the encoded proteins and the cellular significance. (Without effects on them, further studies would be difficult to justify). This is why we used this different, independent test setup. The results showed clearly that Top3B affects mRNAs and proteins encoded by the identified target mRNAs (Fig. 5). This approach should

be more valuable for researchers interested in studying individual Top3B targets than performing the same IPs in more than three biological replicates.

=> We do not see the value of such an experiment. Binding to the RGG motif does not involve covalent interactions. These weaker interactions are generally not stable under our conditions with the ionic detergent SDS. Furthermore, unexpectedly strong interactions with the RGG motif would show the same binding to RGG in the mutant control and the wild type. It would, therefore, show up as a false negative and this is less of a problem than a false positive (because we found enough targets). Please also note that the very high number of different samples (different genotypes, different conditions, and all x3) forced us to focus on the most important controls, which we still think we did.

5. The authors claim that more than 4000 genes are altered more than 2-fold in Top3b-KO flies based on RNA-seq data. We are skeptical about such a large change, because the phenotype of Top3b-KO flies is not very strong. We have tried to download the RNA-seq data deposited at GEO by the authors to check the validity of author's claims. However, these files appear to contain errors, which prevent us from analyzing these data. Possibly, the errors could be generated by disruption in the uploading process, which occur quietly commonly. Can the authors resubmit the their raw to GEO, and make sure that the uploaded files can be downloaded and analyzed?
➔ (Without rounding the data, I now found 2680 genes downregulated in the null mutant (using the less stringent criteria and 1453 with the more stringent ones) according to Fig. 1A). It should be kept in mind that we expect that every RNA can become a Top3B target and, therefore, every gene could already be directly affected. Also, as opposed to the on-off effects one often sees in studying developmental control processes, the reduction of RNA levels can be expected to be less drastic because we can expect that only a fraction of a specific mRNA pool depends on Top3B (e.g., the ones that run into structural problems). Furthermore, it was estimated that in Drosophila, more than half the genes can be knocked down without displaying easily detectable phenotypes and it has been shown that heterozygous deficiencies are mostly viable, suggesting that half the dose of gene products from the deficiency region causes no serious problems in a lab setting. The combination of these three points probably explains why the phenotypes are not more pronounced. I am not "skeptical" about these numbers, I think they fit the process we describe in this manuscript perfectly.

Our Bioinformatics support group usually submits such data to a different database. It was only because this reviewer requested that we submit it to this database. Strangely, the quality control of GEO did not detect the problem. We have now submitted the data again.

There is no reviewer 2 this time.

➔ Reviewer 2 was more positive and had more minor requests and questions in the first round. Did we satisfy this reviewer with our revisions?
