# Supplementary material for: Drosophila Topoisomerase 3β binds to mRNAs in vivo, contributes to their localization and stability, and counteracts premature aging
Source: PLoS One. 2025 Feb 11;20(2):e0318142. doi: 10.1371/journal.pone.0318142 (PMC12140118; doi:10.1371/journal.pone.0318142)
Supplement: Attachment — Submitted filename: renamed_e938c.pdf [file pone.0318142.s024.pdf]

Responses to the Reviewer's comments

6. Review Comments to the Author

Please use the space provided to explain your answers to the questions above. You may also include additional comments for the author, including concerns about dual publication, research ethics, or publication ethics. (Please upload your review as an attachment if it exceeds 20,000 characters)

Reviewer #3: The RNA topoisomerase activity of Top3B in eukaryotes is an interesting topic, and have implications for neurological development and other physiological functions that potentially require such RNA topoisomerase activity. The identification of RNA targets of Drosophila Top3B in early embryonic stage is informative. The alteration of subcellular localization of certain gene products in an active site mutant of Top3B is an important result, suggesting that large genes and long and complex transcripts may need Top3B to be transported and translationally controlled.

Both Ref 74 and data in Fig 1A of this manuscript showed that the active site Tyr point mutation that specifically disrupted Top3B catalytic activity only partially recapitulated the effects of TOP3B-null mutation. Su et al. suggested that Top3B–TDRD3 can regulate mRNA translation and turnover by mechanisms that are dependent and independent of topoisomerase activity. It would be useful for the authors to comment on the possibility of catalytic activity independent mechanism of RNA regulation by Top3B based on the Drosophila data comparing effect of Top3B26 and Top3BY322F mutations described here.
➔ (Because our manuscript focuses on the enzymatic and cytoplasmic functions of Top3B, this point had lower priority in the previous version.) In the revised MS, I added a couple of paragraphs to the first section of the discussion to cover this point. This addition required a reorganization of this first section. Therefore, I highlighted the entire section in the marked-up version.

Reviewer #4: Major comments:

1. The authors show that TOP3B binds long maternal RNAs in a catalytic activity dependent manner in 0-2h embryos. While the absence and mutation of TOP3B leads to severe downregulations of mRNA abundance found, particularly those related to morphogenesis, synapse signaling, transmembrane transport, cell adhesion and cuticle formation. Yet this downregulation in maternal RNA abundance found in embryos does not manifest in developmental phenotypes.

As this transcriptomic analysis captures maternal mRNA from the oocyte and nurse cells, all these findings concretely suggest is that maternal mRNA, or at least large quantities of maternal mRNA is not necessary for embryonic and larval development.
➔ A phenotypic effect of the widespread reduction of mRNA levels in the young embryos is indeed difficult to find. A combination of several reasons seems to account for this. Lack of *Top3β* activity does not abolish mRNA levels completely but reduces levels of different mRNAs to different degrees. The reduction of many different mRNA levels may not be deleterious for embryos because the majority of the Drosophila genes can be knocked down or out without causing visible phenotypes. Furthermore, heterozygous deficiencies are mostly viable, suggesting that half the dose of gene products from many genes in the deficiency regions causes no immediate serious problems in a lab setting.

I now comment this in fewer words in the results section that covers Fig. 3. I also point out that this suboptimal situation most likely stresses the cells. In the subsequent Gene Ontology study I then introduce the idea that the reported molecular defects and cellular stress could have effects later in the life cycle. This section then makes the connection to neurodegeneration.

In the “***Top3β* supports neuronal structure and function in aging adults**” section we had already presented the interpretation that the premature aging in the mutant might be a consequence of the earlier effects of gene expression defects that can be partially compensated during embryogenesis. Also that this partial compensation is likely costly for the cells and stresses them, seeding the later neurodegenerative defects. This mechanism has also been proposed for in human neurodegeneration and cancer.

Overall, the argument for the role of TOP3B in neurodegeneration is weak as transcriptome analysis was only done in embryos.

➔ We agree that we did not directly link the lack of *Top3β* activity to neurodegeneration caused by specific transcripts. Considering that the rapid embryonic development also uses many maternally deposited mRNAs and encoded protein products at later embryonic stages, it seems likely that the reduced early embryonic levels of these mRNAs (Fig. 1) and cellular proteins (Fig. 3) in *Top3β* mutants also cause stress and possibly structural problems during nervous system development. While not showing a direct effect, this explanation is consistent with the hypothesis and the Gene Ontology analysis. A more direct way to show the role of *Top3β* in preventing neurodegeneration came from the analyses of degenerating motoneurons in older flies (new Fig 4B) and its consequences for coordinated movement and aging of flies (new Fig. 4A and 4C). With the reorganization of the manuscript (new Fig. 4 was Fig. 6), this result has now been closely grouped with the effect on the embryonic RNAs and proteins. The closer connections between the different arguments should now strengthen our interpretation. We also discuss what we think is the most likely mechanism that leads to neurodegeneration.

2. While the authors further confirm TOP3B favors binding to long RNAs in Figure 3, this is neither a novel phenomenon nor is it important in neural development and development overall.

➔ In the context of the new Fig. 2A (former Fig. 3A), we used this result as an indication that the quality of the data is good because we saw this preference. We also use this result when discussing possible mechanisms that lead to less mRNA or reduced protein synthesis (because longer linear molecules are more likely to run into structural problems that prevent their use for various steps in gene expression).

It is also confusing when the authors mention that 0-2h embryos reflect the summation of changes in mRNA level from transcription in the oocyte/nurse cells to translation in the embryos (Line 171-173), while also using the lack of zygotic transcription of 0-2h embryos to isolate direct RNA targets of TOP3B (Line 245-246).

➔ I am not entirely sure why this is confusing. Therefore, my explanation and description of the changes that should clarify this might become slightly lengthy. For the effect on the transcriptome (Fig. 1), mothers and embryos have the same *Top3β** genotype, excluding the presence of different Top3β* variants in the embryo. Cellular mRNAs present in young embryos have been synthesized in the nurse cells of their mother during oogenesis. Nurse cells are sister cells of the oocyte, and the two cell types remain connected through cytoplasmic bridges to each other after the precursor cell has divided. Cytoplasmic transport on microtubules imports the mRNAs into the oocyte through these bridges. Top3B might, therefore, modulate the mRNA levels already in the nurse cell nuclei during transcription (through its activity on RNA or DNA), splicing and export, and in the cytoplasm during transport and anchoring in the oocyte. In the young embryo, Top3B could then affect mRNA levels in the cytoplasm during transport, anchoring, and translation. Additionally, some RNA level changes might induce secondary changes in the expression of its own or other genes during oogenesis because transcription is going on in the ovary over several days (allowing also compensatory gene expression and feedback loops).

For the capturing experiment (new Fig 2; previous Fig. 3), we also used the young embryos that do not transcribe yet. We, therefore, expect that there are no pre-mRNAs in the nuclei

and that we capture almost exclusively mature mRNAs in the cytoplasm. As opposed to the other experiment, this work was done in the endogenous *Top3β⁺* background with an additional copy of the Top3β*::GFP variants. In this background, RNAs can be processed normally by the endogenous Top3β, and we can capture them with Top3β::GFP. Because we capture RNAs that are only transiently bound during the enzymatic topoisomerase cycle, we can only capture cytoplasmic RNAs that were made earlier in the mother, deposited into the egg, and bound to Top3β::GFP at the time of lysis. It is important to bring these points across because it explains why we cannot expect to find the same set of hits in the two datasets. I reworked these explanations in the manuscript to make them better accessible to the reader.

The authors also reason that TOP3B is especially necessary for long RNAs that are prone to physical stress to their structure (Line 280-282). With this RNA-IP, do the authors mean to imply that TOP3B dominantly affects gene expression through binding to long RNA to facilitate its localization and translation with negligible effects on DNA and transcription?

➔ No, we performed these RNA-IPs (new Fig. 2; previous Fig. 3) on purpose with material collected from a stage when transcription is insignificant. We wanted to focus specifically on the posttranscriptional activities of the enzyme Top3β in the cytoplasm because less is known about them, and it was the focus of our research interest. This was also reflected in the title of this section "*In vivo* RNA interactions of Top3β in the absence of bulk transcription". It is one of the great things about our model system that it provides the opportunity to study such a situation. We have now made some changes that should emphasize this better. Additionally, we had already mentioned which observed effects could reflect nuclear functions in promoter usage, transcription, splicing, and nuclear export. In the new version, we have added a bit more results and discussion of these other activities on RNAs. However, our studies do not allow us to address its DNA functions.

3. Section on parallels between Drosophila TOP3B/FMR1 and mammalian TOP3B/FMRP does not logically flow with the previous section, and is almost negligible to the manuscript, placing it at the end would be more suitable to understate its low impact to the overall message.
➔ This is a good concept, I reorganized the manuscript as suggested and changed the transitions accordingly where required.

4. Line 364-371 is an excuse with barely any relevance to both the previous section and the next paragraph, which only serves to remind the readers how insignificant TOP3B's role is in embryogenesis and highlights the futility of visualizing RNA localization in embryos and not in neuronal cells. Placing Figure 5 data right after Figure 3 would improve readability.
➔ Good point, this works fine. I reorganized the order as suggested and deleted the "excuse" paragraph. But I used some parts of this paragraph to respond to point 1. With the reorganization, previous Figure 3 became new Figure 2 and previous Figure 5 is now new Figure 3.

5. Figure 6 demonstrates that TOP3B depletion and catalytic mutant finally have phenotypes in age-related degeneration. Yet all explanation behind the mechanism is based on an oocyte transcriptome, and other correlative clues (Figures 2 and 4) to neurodegeneration. The authors should consider a transcriptome analysis of young adult and aged animals to directly show the causative effects of the lack/mutation of TOP3B.
➔ At first sight, it sounds like the thing to do. However, it would be challenging, and the results might be non-informative. Total adults would contain insufficient (periferal) nervous cells, and isolating the peripheral nervous system would be a big challenge. In parallel with the embryonic RNA pull-down analyses, we had performed the same experiments with adult heads. But this gave no conclusive results (presumable because even heads are not uniform enough and contain too much non-neuronal tissue. One of the problems working with a complex animal system with small brains). The other concern is that, like with human

neurodegeneration or cancer, the initial onset of the problem happens probably much earlier. Cellular processes that are not executed perfectly are causing cellular stress and, in the nervous system, subsequent premature aging and neurodegeneration. This is an important point we have tried to bring up in the manuscript. To substantiate this view, the defects in embryonic mRNA and protein expression and localization are important and should not be played down too much. If our interpretation is correct, an analysis of the nervous system transcriptome of adults would only reveal numerous downstream effects and not the most important primary targets.

6. Finally, the authors introduced G4C2 repeats found in human neurodegenerative diseases to TOP3B fly mutants. The authors need to justify a few things: whether or not TOP3B has been shown to suppress toxicity of G4C2 repeats in Drosophila/other species and why the adult eyes specifically was used to assess a motor neurodegenerative disorder stimulated by G4C2 repeats.

➔ We now provide an extended introduction to this experiment where we point out that in addition to testing the effect of Top3β on the normal expression of cellular mRNAs, we wanted to test whether Top3β's catalytic activity also affects the expression and the toxic effect of a repeat-expansion RNA such as the human C9orf72 RNA. We are not aware of experiments that tested the effect of Top3β on G4C2 repeats in this or another system. We also explain that because it is a neuronal system and extremely sensitive (and easy to manipulate the genetic background), it has been developed and used by several labs interested in neurodegeneration and modeling the degeneration of motoneurones.

Minor comments:

1. All titles (main title, result and discussion section titles) are vague and inconclusive.
➔ We have now made changes to titles and subtitles that emphasize the results more than indicating the research topic.

2. Figure 2 and its text section is misplaced and irrelevant to the previous section, as there is nothing in the previous data (Figure 1, Supplementary Figures 1-3) reflecting a relationship between the transcriptome results with neurological disorders and cancer. Figure 2 only becomes relevant when grouped with Figure 6.
➔ We have now found a way to integrate Figure 2 into the suggested flow.

---

7. PLOS authors have the option to publish the peer review history of their article (what does this mean?). If published, this will include your full peer review and any attached files.

If you choose “no”, your identity will remain anonymous but your review may still be made public.
